# The *Arabidopsis* a zinc finger domain protein ARS1 is essential for seed germination and ROS homeostasis in response to ABA and oxidative stress

**DOI:** 10.3389/fpls.2015.00963

**Published:** 2015-11-04

**Authors:** Dongwon Baek, Joon-Yung Cha, Songhwa Kang, Bokyung Park, Hyo-Jung Lee, Hyewon Hong, Hyun Jin Chun, Doh Hoon Kim, Min Chul Kim, Sang Yeol Lee, Dae-Jin Yun

**Affiliations:** ^1^Division of Applied Life Science (BK21 Plus Program), Plant Molecular Biology and Biotechnology Research Center, Gyeongsang National UniversityJinju, South Korea; ^2^College of Life Science and Natural Resources, Dong-A UniversityBusan, South Korea

**Keywords:** abiotic stress, abscisic acid, *Arabidopsis*, C2H2 zinc finger, reactive oxygen species, redox

## Abstract

The phytohormone abscisic acid (ABA) induces accumulation of reactive oxygen species (ROS), which can disrupt seed dormancy and plant development. Here, we report the isolation and characterization of an *Arabidopsis thaliana* mutant called *ars1* (*aba and ros sensitive 1*) that showed hypersensitivity to ABA during seed germination and to methyl viologen (MV) at the seedling stage. *ARS1* encodes a nuclear protein with one zinc finger domain, two nuclear localization signal (NLS) domains, and one nuclear export signal (NES). The *ars1* mutants showed reduced expression of a gene for superoxide dismutase (*CSD3*) and enhanced accumulation of ROS after ABA treatment. Transient expression of ARS1 in *Arabidopsis* protoplasts strongly suppressed ABA-mediated ROS production. Interestingly, nuclear-localized ARS1 translocated to the cytoplasm in response to treatment with ABA, H_2_O_2_, or MV. Taken together, these results suggest that ARS1 modulates seed germination and ROS homeostasis in response to ABA and oxidative stress in plants.

## Introduction

The phytohormone abscisic acid (ABA) regulates important physiological processes including embryogenesis, seed dormancy, vegetative growth, and abiotic stress responses (Cutler et al., 2010; Raghavendra et al., 2010). ABA signaling is associated with the accumulation of intracellular reactive oxygen species (ROS), which initiates diverse signal transduction processes such as gene expression, enzyme activation, and programmed cell death (Neill et al., 2002; Wasilewska et al., 2008). ROS such as hydrogen peroxide (H_2_O_2_), superoxide anion (O_2_^-^), singlet oxygen (^1^O_2_), and hydroxyl radical (OH^-^) form as toxic byproducts of metabolic processes, including photosynthesis, dark respiration, and photorespiration, as well as under abiotic stress conditions; ROS also act as important signaling molecules under optimal growth conditions (Mittler et al., 2004; Kim et al., 2008; Foyer and Shigeoka, 2011).

Reactive oxygen species, as key endogenous messengers, play a crucial role in the complex ABA signaling network (Wang and Song, 2008). This network involves diverse regulators, such as NADPH oxidases, SNF1-related protein kinases (SnRK), type-2C/A protein phosphatases (PP2C), calcineurin B-like (CBL) interacting protein kinases (CIPK), calcium-dependent protein kinases, and mitogen-activated protein kinases (MAPK). The ABA receptors RCAR/PYR1/PYL (Regulatory Components of ABA-receptor/Pyrabactin resistant Protein/PYR-like protein) perceive ABA, bind to ABA, and interact with a group of PP2Cs (Ma et al., 2009; Park et al., 2009). In the absence of ABA, PP2Cs interact with SnRK2/OST1 (OPEN STOMATA1) and dephosphorylate SnRK2 to inactive its kinase activity. When ABA binds to its receptors, SnRK2 is activated, via the lack of PP2C function (Umezawa et al., 2009). Activated SnRK2 phosphorylates and regulates various downstream target proteins, including guard cell ion channels, NADPH oxidase, and transcription factors (Kwak et al., 2003; Sato et al., 2009; Yoshida et al., 2010; Brandt et al., 2012). Among the SnRK2 targets, NADPH oxidases (respiratory burst oxidase homologues, RBOHs) localize in the plasma membrane and the phosphorylation of RBOHs by SnRK2/OST1 plays a major role in triggering ROS production in plants (Kwak et al., 2003). In addition, RBOHF is phosphorylated by the CBL/CBL9-CIPK26 complex and mediates ROS production (Drerup et al., 2013).

Regulation of ROS-generating and ROS-scavenging systems maintains the delicate balance of ROS (Foyer and Noctor, 2009). Non-enzymatic antioxidants detoxify singlet oxygen and hydroxyl radical; by contrast, antioxidant enzymes including superoxide dismutases (SOD), catalases (CAT), and ascorbate peroxidases (APX) detoxify H_2_O_2_ (Op den Camp et al., 2003; Gadjev et al., 2006; Gechev et al., 2006; Foyer and Noctor, 2009). SOD catalyzes the dismutation of O_2_^-^ to H_2_O_2_; CAT and APX directly react with H_2_O_2_ to form water and oxygen (Mittler et al., 2004).

Recently, ROS have been implicated in mediating complex, systemic signaling in plant cells. These ROS signals may function alone or interact with other molecules, including plant hormones, Ca^2+^ signals, proteins, and RNA (Karpiński et al., 2013; Shah et al., 2014; Wang et al., 2014). In addition, ABA and ROS may act together in regulating systemic responses to abiotic stress (Suzuki et al., 2013). In this study, we isolated *Arabidopsis ARS1* (*ABA AND ROS SENSITIVE 1*) using a genetic screening system and showed that ARS1 is essential for seed germination and maintenance of ROS homeostasis in plants challenged with ABA or oxidative stress. We also demonstrated that ARS1 translocates from the nucleus to the cytoplasm in response to ABA or oxidative stress. We report that ARS1 functions as a positive regulator countering ABA to break seed dormancy and maintaining ROS homeostasis in response to ABA and oxidative stress.

## Materials and Methods

### Plant Materials and Growth Conditions

The activation T-DNA vector *pSKI015* was used to generate an insertion mutant population (T1) in the *Arabidopsis thaliana* C24 *RD29A::LUC* (WT) background, based on Basta herbicide selection. Plants were grouped into 10-line pools, and T2 progenies were screened for mutants that exhibited ABA hypersensitivity compared to WT. T-DNA insertion mutants of *ARS1* (*At3g02860*), *ars1-2* (SALK_009596), *ars1-3* (SALK_030445), and *ars1-4* (SALK_126300), were obtained from the *Arabidopsis* Biological Resource Center (ABRC). Seeds were surface-sterilized and sown onto MS medium [1/2 Murashige and Skoog (MS) salts, 1.5% sucrose and 0.6% agar, pH 5.7] with or without ABA (as indicated in figures). Plants were grown in a growth chamber with a cycle of 16 h light and 8 h dark at 22°C

### Thermal Asymmetric Interlaced PCR Analysis

DNA flanking the left border of the inserted T-DNA in the *ars1-1* mutant was isolated by thermal asymmetric interlaced PCR (TAIL-PCR). The entire isolated fragment was sequenced. The primers used in the TAIL-PCR were specific primers for the T-DNA left border (LB1, LB2, and LB3) and degenerate primers (DP1, DP2, and DP3; Supplementary Table S1). The nucleotide sequence of the PCR product was determined and subjected to BLASTn analysis.

### Determination of Transcript Levels

Total RNA was isolated from 10-d-old seedlings using the RNeasy kit (Qiagen, Valencia, CA, USA) following the manufacturer’s instructions. Isolated total RNA was treated with DNase I (Qiagen, Valencia, CA, USA) to remove genomic DNA contamination. The first-strand cDNA was synthesized using 2 μg total RNA with a cDNA synthesis kit (Invitrogen, Carlsbad, CA, USA) in a 20-μl reaction volume, and subjected to PCR for examination of gene expression. The specific primers were designed according to the sequence of *ARS1*. *TUBULIN2* was used as a control in the experiment. The primers used for the RT-PCR analysis are listed in Supplementary Table S1.

For quantitative RT-PCR (qRT-PCR) analysis, the first-strand cDNA was synthesized using 2 μg total RNA with a cDNA synthesis kit (Invitrogen, Carlsbad, CA, USA). The QuantiSpeed SYBR No-Rox Mix (PhileKorea, Seoul, Korea) was used for qRT-PCR as follows: 50°C for 10 min, 95°C for 2 min, and 40 cycles of 95°C for 5 s, and 60°C for 30 s. *TUBULIN2* was used for RNA normalization. The relative expression levels of all samples were automatically calculated using CFX Manager program (Bio-Rad, Hercules, CA, USA) and carried out in three biological replicates. The primers used for the qRT-PCR analysis are listed in Supplementary Table S1.

### Protoplast Transient Expression Analysis

The cDNA encoding *ARS1* was isolated from a cDNA library by PCR. The PCR product was confirmed by nucleotide sequencing and was inserted into *Xba*I and *Bam*HI sites of the *sGFP* vector (kindly provided by Inhwan Hwang, POSTEC, Korea) to create chimeric GFP-fusion constructs under the control of the *35S* promoter (Supplementary Table S1). The *sGFP* plasmid vector is a *pUC*-based vector containing *CaMV35S-sGFP-NOS3* for protoplast expression.

Protoplast isolation from *Arabidopsis* leaves and transformation into protoplasts was as described in Jin et al. (2001). Expression of the fusion constructs was monitored at various time points after transformation and images were captured with a Zeiss Axioplan fluorescence microscope (Carl Zeiss Co., Jena, Germany). The filter sets used were: XF116 (exciter, 474AF20; dichroic, 500DRLP; and emitter, 605DF50) and XF137 (exciter, 540AF30; dichroic, 500DRLP; and emitter, 585ALP; Omega, Inc., Brattleboro, VT) for GFP and RFP, respectively. Data were then processed using Adobe Photoshop software (Adobe System, Mountainview, CA, USA) and presented in pseudo-color format.

### Detection of ROS in Protoplasts

To measure intracellular ROS levels, an aliquot of protoplast suspension (∼2 × 10^5^⋅ml^-1^) was incubated with 5 μM 2,7-dichlorohydrofluoroscein diacetate (DCFH-DA, Molecular Probes, Eugene, OR, USA) for 5 min and 20 μM dihydrorhodamine123 (Rh123, Molecular Probes, Eugene, OR, USA) for 15 min and were observed under a Zeiss Axioplan fluorescence microscope using XF116 (DCFH-DA; exciter, 474AF20; dichroic, 500DRLP; and emitter, 605DF50) and XF33/E (Rh123; exciter, 535DF35; dichroic, 570DRLP; emitter, 605DF50; Omega, Inc., Brattleboro, VT, USA).

### Histochemical Detection of O_2_^-^

NBT (nitro blue tetrazolium; Sigma–Aldrich, Saint Louis, MO, USA) staining was used to detect O_2_^-^ accumulation in tissues. O_2_^-^ was visualized as a dark blue formazan compound within tissues. Seven-day-old seedlings were immersed in 50 mM potassium phosphate buffer (pH 7.8) containing 0.1% NBT and 10 mM sodium azide and incubated for 2 h in the dark. Chlorophyll was removed from the seedlings prior to imaging by infiltrating them with lacto-glycerol-ethanol (1: 1: 4 volume) and boiling for 5 min (Bournonville and Díaz-Ricci, 2011).

### Measurement of H_2_O_2_

H_2_O_2_ was measured in tissues using the Amplex Red Hydrogen Peroxide/Peroxidase Assay Kit (Invitrogen/Molecular Probes, Eugene, OR, USA) following the manufacturer’s instructions. Fluorescence was determined by excitation at 530 nm and emission at 590 nm. H_2_O_2_ concentration was calculated based on a standard curve and expressed as H_2_O_2_ per fresh weight (Bournonville and Díaz-Ricci, 2011).

## Results

### Isolation and Identification of the ABA-Hypersensitive *ars1* Mutant

The *RD29Apro::LUC* transgene has been widely adapted to screen for ABA- or abiotic stress-responsive mutants from large populations of *Arabidopsis* C24 ecotype plants with T-DNA insertions (Ishitani et al., 1997; Zhu et al., 2008). Here, the T_2_ progeny were screened for enhanced sensitivity of seed germination to ABA compared to wild type (WT, C24 ecotype), and the phenotypes were further identified in T_3_ progeny. The *ars1-1* mutant displayed hypersensitivity to ABA with reduced seed germination and retarded emergence of green cotyledons compared to WT (Supplementary Figure S1). Using TAIL-PCR, we found that the *ars1-1* mutant had a T-DNA insertion in *ARS1* (At3g02860), located in the second exon, 701 bp upstream of the ATG translation start site. Genotypic analysis showed the T-DNA insertion in *ARS1* segregated with the *ars1-1* mutant phenotype, as all tested F_2_ homozygotes exhibited identical phenotypes (*n* = 100, data not shown).

**FIGURE 1:**
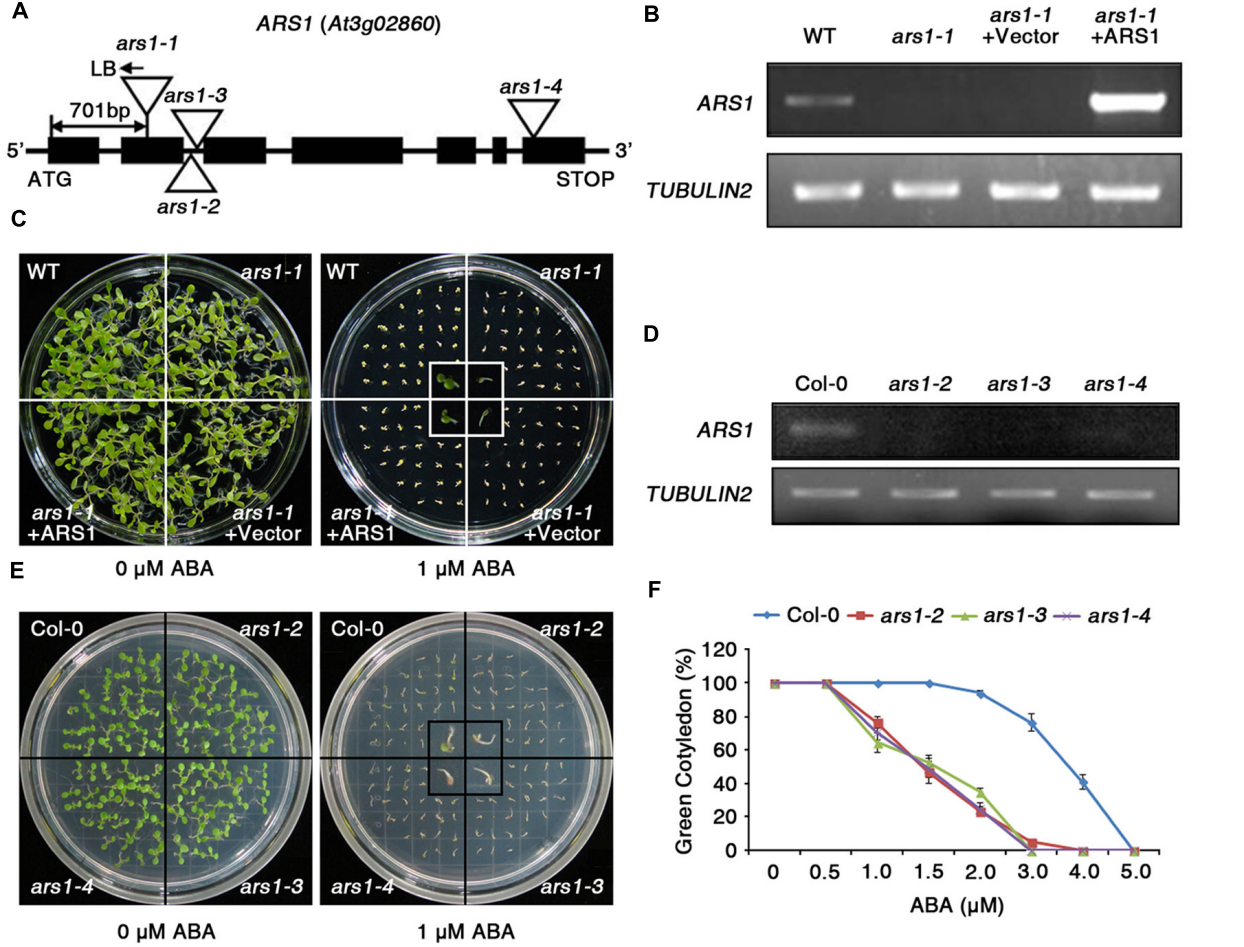
**The absence of *ARS1* leads to ABA-hypersensitive seed germination. (A)** T-DNA insertions in the *ars1* mutant alleles. **(B)** RT-PCR analysis of *ARS1* expression in WT, *ars1-1*, *ars1-1*+Vector, and *ars1-1*+*ARS1* plants. *TUBULIN2* serves as a control for RNA integrity. **(C)** Comparison of seed germination between the WT, *ars1-1*, *ars1-1*+Vector, and *ars1-1*+*ARS1* plants exposed to 0 or 1 μM ABA. The photograph shows *Arabidopsis* seedlings after 5 days of ABA treatment. **(D)** Expression of *ARS1* in WT Col-0 and *ars1* allelic mutants determined by RT-PCR. *TUBULIN2* serves as a control for RNA integrity. **(E)** Comparison of seed germination between the WT Col-0 and *ars1* mutants exposed to 0 or 1 μM ABA. The photograph shows *Arabidopsis* seedlings after 5 days of ABA treatment. **(F)** Quantification of green cotyledons in WT Col-0 and *ars1* mutants grown on various concentrations of ABA for 5 days. The data represent the means ± SE of three independent experiments, with 50 seeds per experiment.

*ARS1* encodes a zinc ion-binding protein (**Figure 1A**), but its biological functions have not been reported yet. To elucidate the functional roles of *ARS1* in the ABA response, we generated a construct overexpressing *ARS1* under the control of the cauliflower mosaic virus *35S* promoter, and transformed this construct into the *ars1-1* mutant (*ars1-1*+*ARS1*). RT-PCR analysis revealed that the *ars1-1*+*ARS1* plants had high levels of *ARS1* transcripts compared to WT, but *ARS1* transcripts were absent in both *ars1-1* and *ars1-1* harboring the empty vector (*ars1-1*+Vector) (**Figure 1B**). In addition, *ars1-1*+*ARS1* plants showed rescue of the ABA hypersensitivity of *ars1-1* and displayed WT phenotypes in seed germination, while *ars1-1*+Vector showed germination defects similar to those of *ars1-1* (**Figure 1C**).

To identify the roles of *ARS1* in response to ABA in multiple alleles, we obtained three additional, different T-DNA alleles, *ars1-2*, *ars1-3*, and *ars1-4*, in the *Arabidopsis* Col-0 background (**Figure 1A**). *ARS1* transcripts were absent in *ars1-2* and *ars1-3* plants compared to Col-0 plants, but were barely detectable in *ars1-4* (**Figure 1D**). All *ars1* allelic mutants in the Col-0 background also showed an ABA-sensitive phenotype with retarded emergence of green cotyledons compared to Col-0 plants (**Figures 1E,F**). Root length also provided a measure of ABA sensitivity (Supplementary Figure S2). Four-day-old plants grown on MS medium were transferred to MS medium containing ABA, and root growth was monitored 11 days later. At 30 and 40 μM ABA, root growth in *ars1-2* and *ars1-3* plants lacking *ARS1* transcripts was significantly lower compared to that in Col-0 plant; however *ars1-4* mutants showed no significant differences from Col-0. Although the ABA-dependent root growth phenotypes of *ars1-4* marginally differed from the germination phenotypes, the data are consistent with the differences in expression of *ARS1* in those mutants, suggesting that ARS1 may also affect post-germination plant growth (**Figure 1D**, Supplementary Figure S2). These results indicate that ARS1 is essential for breaking seed dormancy in germination, which is inhibited mainly by ABA.

### Characterization of ARS1

**FIGURE 2:**
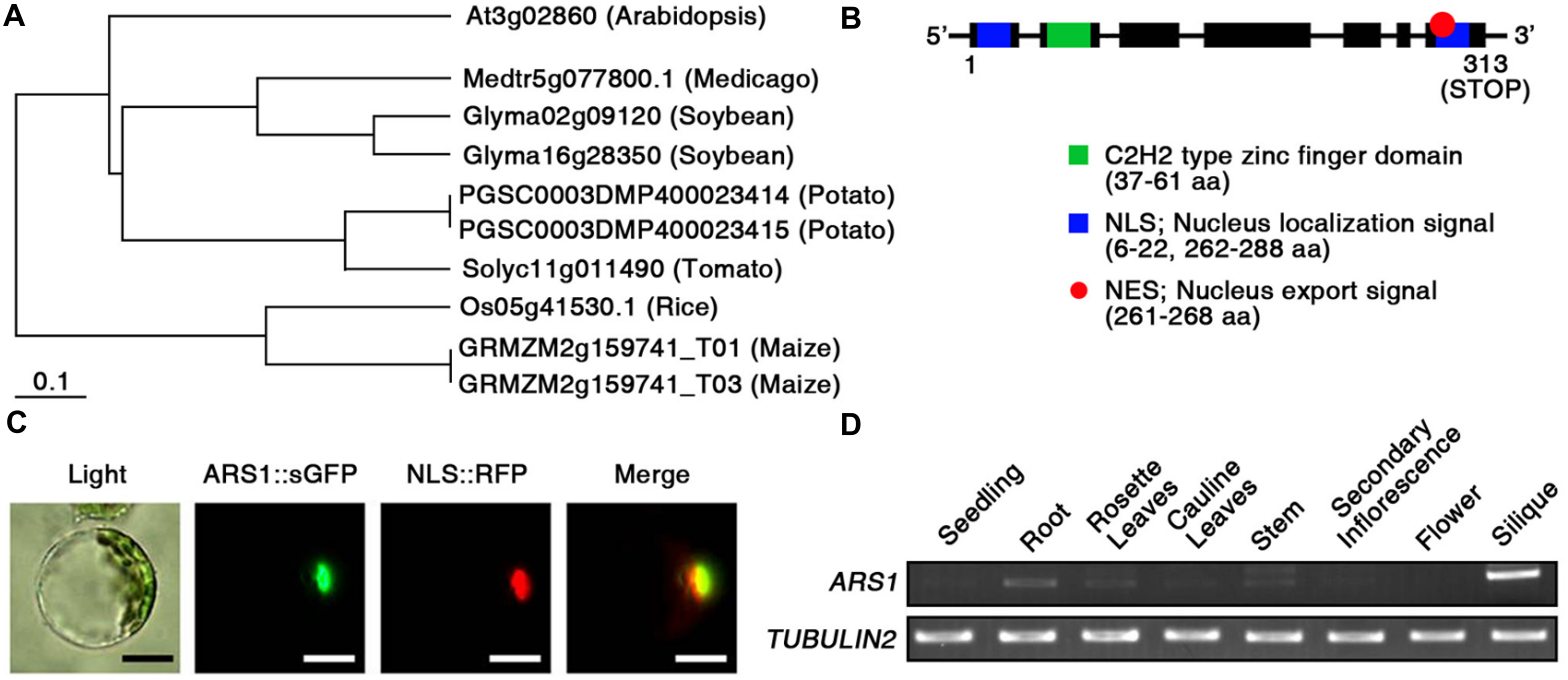
**Characterization of ARS1. (A)** Phylogenetic tree of ARS1 homologs, generated using the BAR Expressolog Tree program. **(B)** *ARS1* encodes an unknown protein with two NLS domains, an NES domain, and a C2H2 type zinc-finger domain. **(C)** Intracellular localization of the ARS1 protein in the nucleus. Protoplasts prepared from *Arabidopsis* seedlings were co-transformed with *ARS1::sGFP* and *NLS::RFP*. The transformed protoplasts were examined by fluorescence microscopy 12 h after transformation. Green and red images are GFP and RFP signals, respectively. Bars indicate 20 μm. **(D)** RT-PCR analysis of *ARS1* expression in *Arabidopsis* tissues. *TUBULIN2* serves as a control for RNA integrity.

To predict the potential function of ARS1 in plants, we compared the protein sequences of *Arabidopsis* ARS1 and its homologs in other plants, retrieved by BLAST-P search. Phylogenetic analysis using the BAR Expressolog Tree program^1^ revealed that ARS1 had the highest sequence similarity (53.1%) to a zinc finger protein in *Medicago truncatula*, and also shared 32.4–49.9% similarity with its homologs in plant species such as *Glycine max*, *Solanum tuberosum*, *Lycopersicon esculentum*, *Oryza sativa*, and *Zea mays* (**Figure 2A**). The tree clearly divided ARS1 homologs from dicots or monocots into different branches. However, their biological functions remain elusive in plants. *ARS1* encoded a 313-amino acid protein with predicted molecular mass of 35.2 kDa^2^ (**Figure 2B**). ARS1 protein possesses a nuclear localization signal (NLS; 6–22 a.a.), a C2H2-type zinc finger domain (37–61 a.a.) in the N-terminal region, a nuclear export signal (NES; 261–268 a.a.), and another NLS (262–288 a.a.) in the C-terminus. C2H2 zinc finger proteins are classified three sets (A, B, and C), and set C is sub-classified into three distinguishable subsets such as C1, C2, and C3 (Englbrecht et al., 2004). We found that ARS1 belongs to subfamily of C3 subset (Supplementary Figure S3). It indicates that ARS1 as a C2H2 zinc finger protein may be located either in the nucleus or cytoplasm and may translocate under certain conditions. To reveal the intracellular localization of ARS1, we used protoplast transient expression co-transforming an *ARS1::sGFP* construct and a chimeric construct containing the NLS domain fused to RFP (*NLS::RFP*) as a nuclear marker (Lee et al., 2001). As shown in **Figure 2C**, the intracellular distribution of green and red fluorescent signals overlapped, indicating that ARS1 localizes to the nucleus. We also examined the expression of *ARS1* transcripts in different organs by RT-PCR analysis (**Figure 2D**). Interestingly, *ARS1* transcripts accumulated to high levels in silique and root, which show strong effects of ABA. *ARS1* expression was slightly higher in rosette leaves and stem than in highly proliferating organs such as seedlings, secondary inflorescences, and flowers.

### ARS1 Represses ABA-Induced ROS Production

Abscisic acid shows a strong relationship with abiotic stress tolerance, especially with drought tolerance via regulation of stomatal closure to reduce water loss (Lim et al., 2015). Thus, we first examined water loss, but found that water loss assays showed no significant differences between Col-0 and *ars1-2* plants in the absence or presence of ABA (Supplementary Figure S4).

**FIGURE 3:**
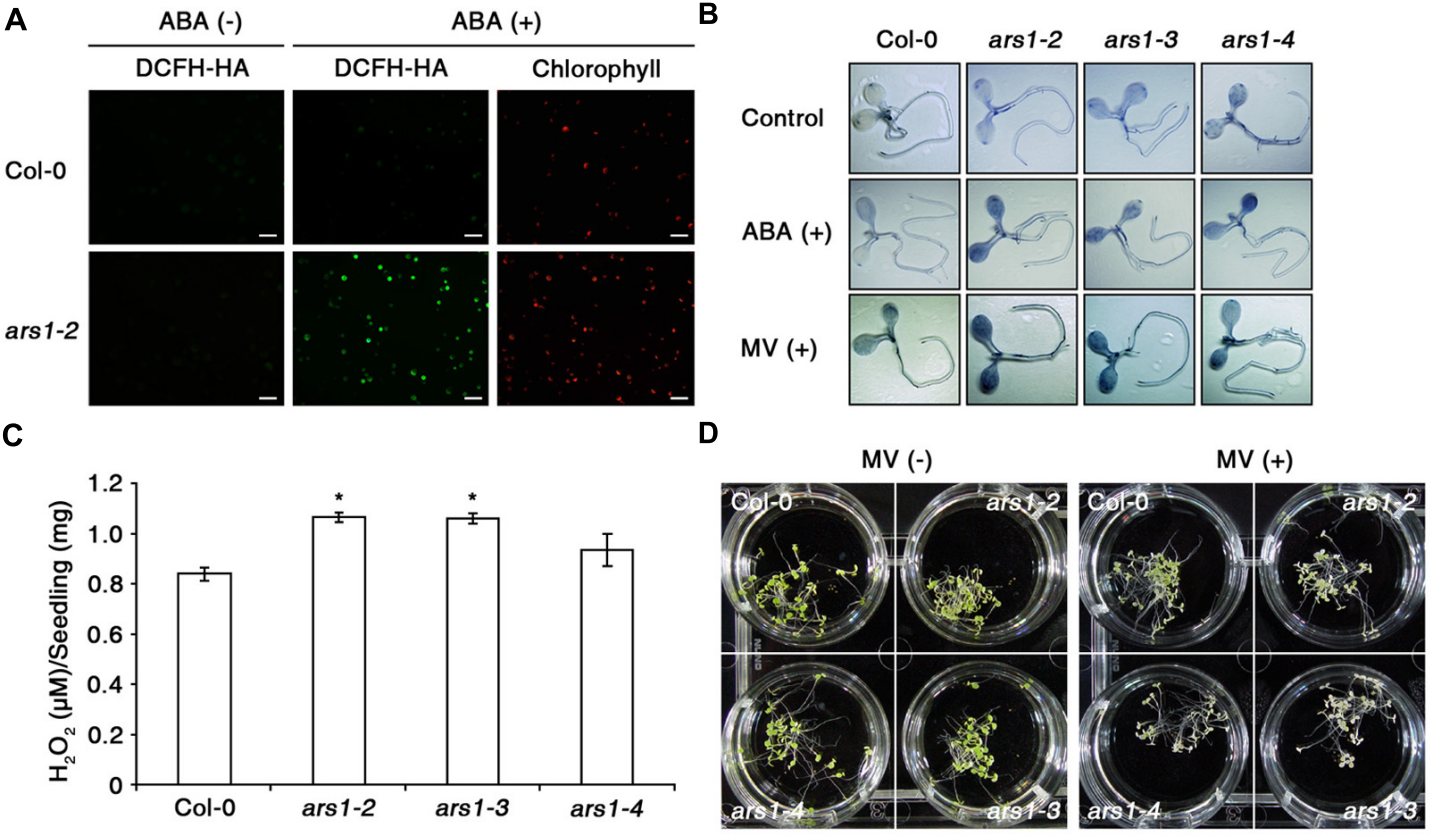
**ARS1 represses ABA-induced ROS production. (A)** Representative images of ROS production indicated by the fluorescent dye DCFH-DA. Protoplasts from 3 week-old-seedlings of WT Col-0 and *ars1-2* mutants were treated with ABA (100 μM) for 1 h. Bars indicate 100 μm. **(B)** WT Col-0 and *ars1* mutants seedlings under ABA (100 μM) and MV (5 μM) treatment stained with NBT. The dark blue color indicates insoluble formazan deposits that are produced when NBT reacts with superoxide. **(C)** Internal H_2_O_2_ production assays in WT Col-0 and *ars1* mutants. Experiments were performed twice, and approximate fluorescence was measured by excitation at 530 nm and emission at 590 nm. Asterisks represent significant differences from the WT (^∗^*p*-value ≤0.05, Student’s *t*-test). **(D)** Photographs of seedlings that were grown on MS medium for 4 days, and transferred to MS medium with or without MV (5 μM) for another 3 days.

Next, we examined ABA-induced ROS production in Col-0 and *ars1-2* protoplasts using an ROS-sensitive, cell-permeable fluorescent dye, 2′,7′-dichlorofluorescin diacetate (DCFH-DA) (Wang and Joseph, 1999; Pei et al., 2000). As shown in **Figure 3A**, the fluorescent signals increased slightly in response to ABA treatment in Col-0 plants, consistent with previous reports (Wang and Song, 2008). Interestingly, the signals increased dramatically in *ars1-2* plants exposed to ABA. In addition, ABA-induced ROS production in *ars1-3* and *ars1-4* was similar to that in *ars1-2* mutants (data not shown). We further examined ABA- or MV- induced ROS induction *in planta* by staining the superoxide anion with nitroblue tetrazolium (NBT) (**Figure 3B**). Both ABA and MV increased the dark purple staining, indicating increased superoxide levels in all *ars1* mutant plants, while those in Col-0 plants remained consistent in the absence or presence of ABA or MV. Interestingly, *ars1* mutants showed more staining than Col-0 plants, even in the absence of treatments, indicating that the absence of ARS1 may increase ROS levels in plants. To confirm the ROS accumulation in *ars1* mutants, we compared hydrogen peroxide contents of Col-0 and *ars1* mutants (**Figure 3C**). As shown in **Figure 3B**, hydrogen peroxide contents in all *ars1* mutants were higher than those in Col-0 plants, even in the absence of treatments. The *ars1-2* and *ars1-3* mutants showed significantly higher ROS than *ars1-4*, which is also consistent with the different levels of *ARS1* transcripts among *ars1* mutants (**Figure 1D**). Furthermore, *ars1* mutants displayed MV-sensitive phenotypes, showing bleaching of leaves compared to Col-0 (**Figure 3D**).

**FIGURE 4:**
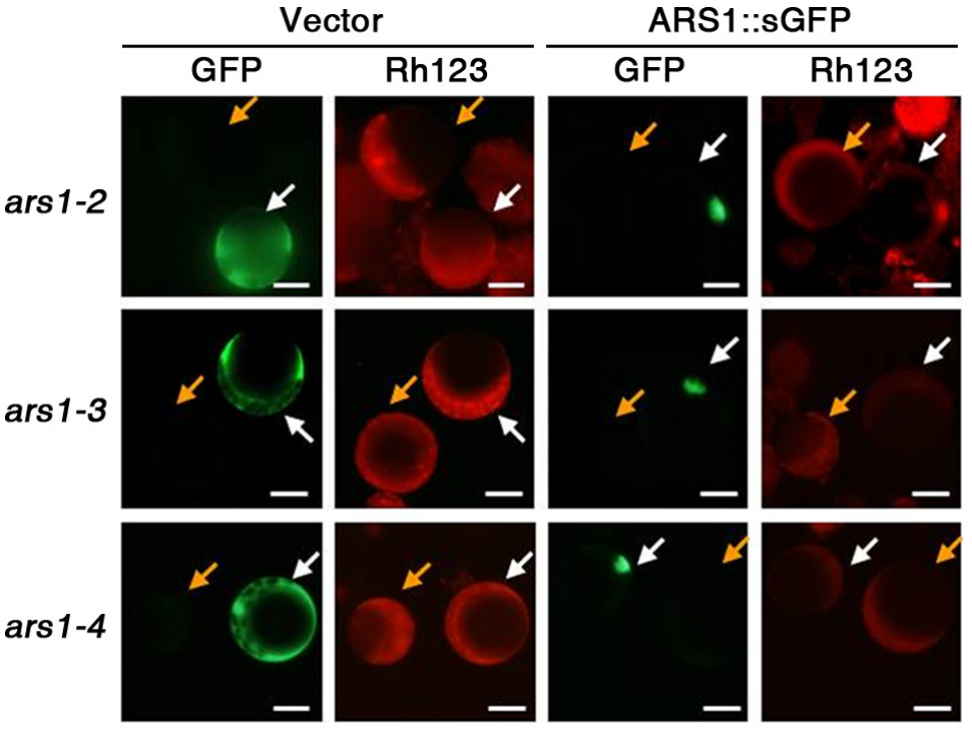
**ARS1 inhibits ABA-induced ROS production.** Protoplasts were isolated from *ars1* mutants seedlings transformed with the empty sGFP vector (Vector) or *ARS1::sGFP*. Twelve hours after transformation, protoplasts were treated with ABA (100 μM) for 1 h and stained with dihydrorhodamine123 (Rh123) for 15 min. The images are green (GFP) and red (Rh123) fluorescence images of one aliquot of protoplasts. Bars indicate 20 μm. Yellow arrows point to the non-expressing protoplasts, white arrows point to the protoplasts expressing sGFP and ARS1::sGFP.

To determine whether ARS1 directly affects ABA-induced ROS production, we carried out complementation tests in protoplasts using the fluorescent dye dihydrorhodamine123 (Rh123) to monitor ROS production. Rh123 becomes the fluorescent chromophore Rh123 upon oxidation by ROS (Schulz et al., 1996). Empty *GFP* vector and *ARS1::sGFP* constructs were independently transformed into protoplasts isolated from the *ars1* mutants, and approximately 40% of the protoplasts showed expression of both constructs (data not shown). After treatment with ABA for 1 h and then with Rh123 for 15 min, we found that *ARS1::sGFP* clearly targeted to the nucleus but the empty vector produced a GFP signal in the cytoplasm of protoplasts from all *ars1* mutants (**Figure 4**). In addition, the fluorescent signals from Rh123 were greatly reduced in all protoplasts transformed with *ARS1::sGFP* compared to those transformed with empty vector (**Figure 4**). These results suggest that ARS1 acts positively in repressing ABA-induced ROS production.

### ARS1 Deficiency Reduces Expression of *SOD*

To identify how ARS1 regulates ABA responses, we first used qRT-PCR to analyze the transcript levels of *ARS1* in response to ABA. *ARS1* transcripts slightly increased (1.2-fold induction) in response to ABA treatments for 1 and 3 h (Supplementary Figure S5A). Englbrecht et al. (2004) found that C2H2 zinc finger proteins act as transcriptional regulators in conserved biological processes in response to stress. Accordingly, we examined the expression of *RD29A* genes in *ars1* mutants to explore the possible role of *ARS1* in the ABA signaling pathway. *RD29A* expression is highly induced by ABA as a stress-responsive marker gene but does not change in response to H_2_O_2_ treatment (Trouverie et al., 2008). However, *RD29A* transcript accumulation did not show any significant differences in the *ars1* mutants compared to that in Col-0 plants either in the absence or presence of ABA (Supplementary Figure S5B). Thus, ARS1 activity may not be necessary for regulation at the transcriptional level in the ABA signaling pathway.

**FIGURE 5:**
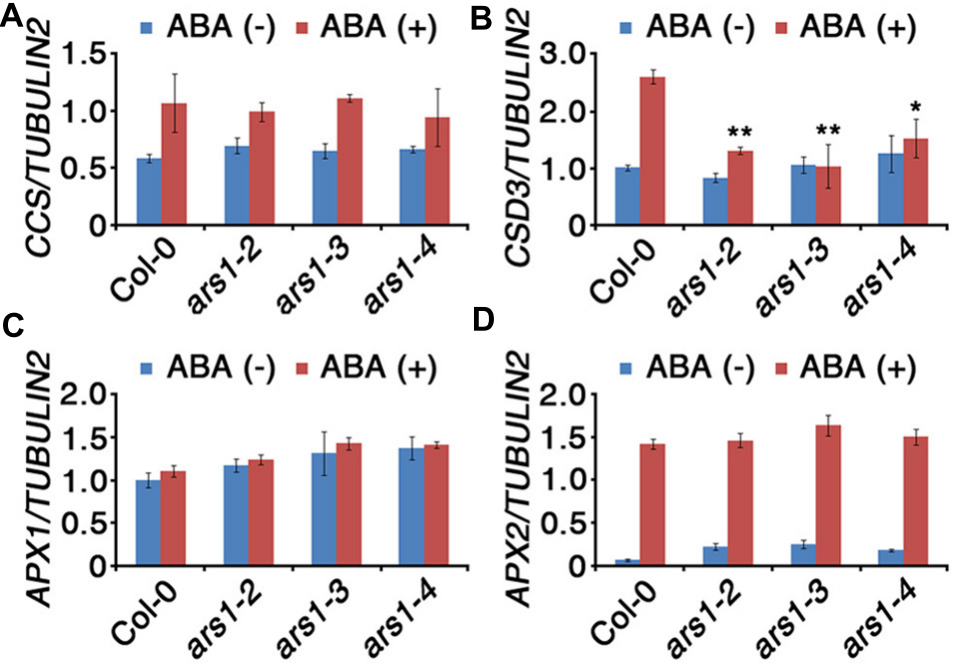
**Expression of ROS-scavenging genes in response to ABA treatment.** Total RNA was isolated from 10-d-old seedlings of WT and *ars1* mutants with or without ABA (100 μM) treatment for 3 h. Relative transcript levels of *CCS* **(A)**, *CSD3* **(B)**, *APX1* **(C)**, and *APX2* **(D)** in Col-0 and *ars1* mutants determined by qRT-PCR. Transcript levels were normalized to those of *TUBULIN2*. Bars represent mean ± SD of three biological replicates with three technical replicates each. Asterisks represent significant differences from the Col-0 (^∗^; 0.01 < *p*-value ≤ 0.05, ^∗∗^; *p*-value ≤ 0.01, Student’s *t*-test).

Based on the effect of ARS1 on ABA-induced ROS production, we investigated the transcripts encoding ROS-scavenging enzymes in *ars1* mutants (**Figure 5**). SOD catalyze the dismutation of O_2_^-^ to O_2_ and H_2_O_2_, which is subsequently reduced to water by CAT and APX (Mittler et al., 2004). We used qRT-PCR to measure the transcript levels of two SOD genes (*CCS* and *CSD3*) and two APX genes (*APX1* and *APX2*) in the absence or presence of ABA. Transcripts of *CCS* (*COPPER/ZINC SUPEROXIDE DISMUTASE*), *APX1*, and *APX2* did not show any significant differences between Col-0 and *ars1* mutants and even in response to ABA treatment (**Figures 5A,C,D**). However, transcripts of *CSD3*, encoding a copper/zinc superoxide dismutase 3, significantly decreased in all *ars1* mutants in response to ABA treatment (**Figure 5B**). These results indicate that ARS1 represses ABA-induced ROS accumulation via inhibiting SOD transcripts, especially *CSD3*.

### ARS1 Translocates from the Nucleus to the Cytoplasm in response to ABA and Oxidative Stress

**FIGURE 6:**
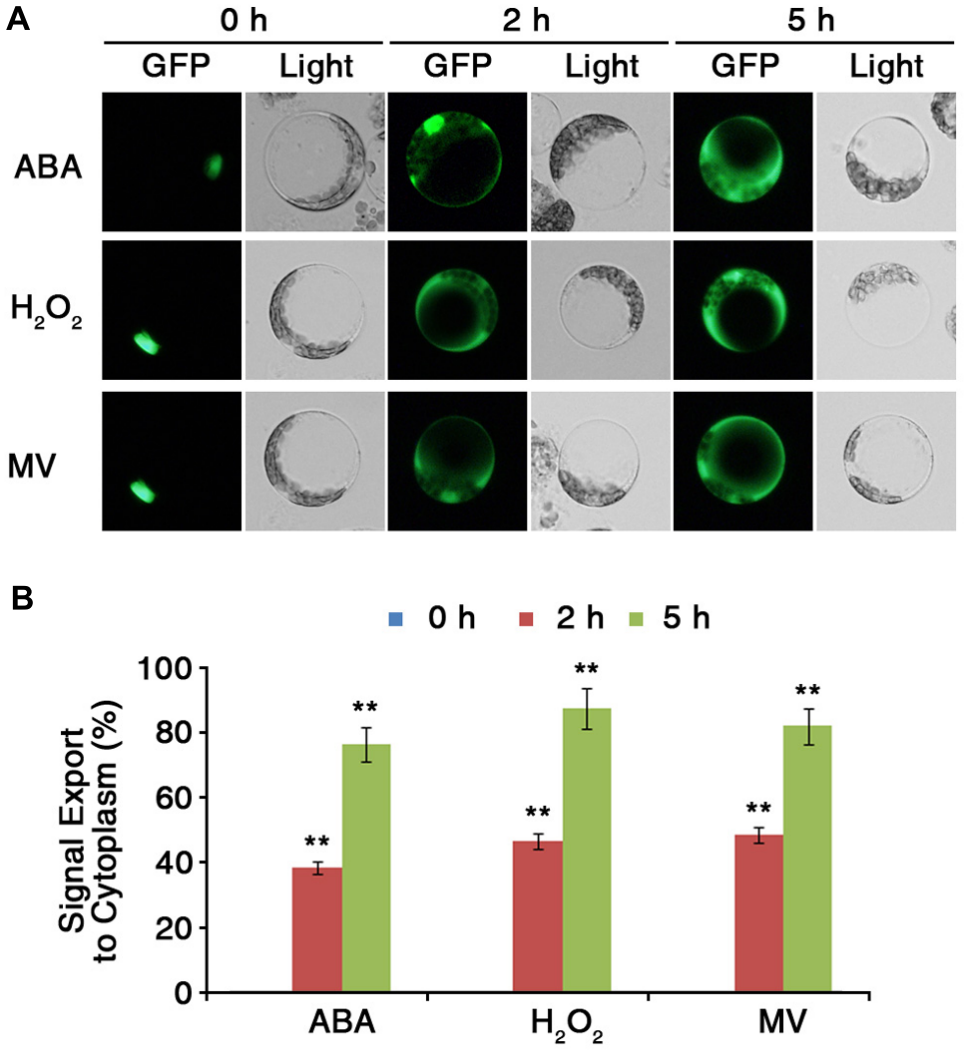
**ARS1 is translocated from the nucleus to the cytoplasm in response to ABA and oxidative stresses. (A)** Protoplasts were isolated from leaves of 3-week-old WT Col-0 plants transformed with *ARS1::sGFP*. Twelve hours after transformation, protoplasts were treated with ABA (100 μM), H_2_O_2_ (1.5 mM) or MV (3 μM) for the indicated times. Bars indicate 20 μm. **(B)** Percentage of ARS1 signal exported to the cytoplasm in response to ABA, H_2_O_2_, and MV shown in **(A)**. Bars represent mean ± SD of three biological replicates with three technical replicates each (*n* = 200). Asterisks represent significant differences from the 0 h(^∗∗^*p*-value ≤0.01, Student’s *t*-test).

Protein sequence analysis revealed that ARS1 has a putative NES motif at its C terminus (**Figure 2B**). The presence of the putative NES signal suggested that ARS1 could be exported from the nucleus in response to certain stress conditions and led us to investigate the changes of subcellular localization of ARS1 in *Arabidopsis* cells. To analyze the subcellular localization of ARS1 under stress conditions, we first transiently expressed *ARS1::sGFP* in *Arabidopsis* (Col-0) protoplast cells. Twelve hours after transformation of *ARS1::sGFP* into protoplasts, we treated the protoplasts with ABA, H_2_O_2_, or methyl viologen (MV) as ROS triggers. In the absence of stressors, ARS1 clearly localized to the nucleus (**Figures 2C** and **6A**, Supplementary Figure S5). Surprisingly, nuclear-localized ARS1 was abundantly translocated to the cytoplasm in response to ABA, H_2_O_2_, and MV. (**Figure 6**, Supplementary Figure S6). These translocation patterns of ARS1 in protoplasts markedly increased as the stress duration increased up to 5 h (**Figure 6B**). These results indicate that the putative NES motif is likely important for ARS1 function in stress responses. Together, this evidence suggests that ARS1 exists as an inactive form in the nucleus, but changes its localization to the cytoplasm as a result of ROS signals induced by ABA and other stresses to repress ABA/stress-induced ROS production in plant cells.

## Discussion

Seed germination involves extensive crosstalk between phytohormones and secondary messengers. ABA and gibberellin (GA) act antagonistically in germinating seeds, and secondary messengers such as ROS and Ca^2+^ might be also responding differently in germination. In dormant seeds, high levels of ABA in seed coats repress germination via expression of the DELLA gene *RGL2* and the ABA biosynthesis gene *ABA1*, thus triggering induction of *ABI5* (Lee et al., 2010). Seed dormancy can be broken by environmental cues such as exposure to cold, oxygen, and water (Finch-Savage and Leubner-Metzger, 2006). In germinating seeds, endogenous ROS and cytosolic Ca^2+^ concentrations increase to promote germination, counteracting the effects of ABA. Exogenous ROS and Ca^2+^ treatments also enhance seed germination (El-Maarouf-Bouteau and Bailly, 2008; Kong et al., 2015). However, either low or excess ROS delay or inhibit germination, indicating that ROS homeostasis, termed the “oxidative window of germination,” is essential for breaking seed dormancy (Bailly et al., 2008). In this study, we identified *ARS1*, encoding an uncharacterized zinc finger domain protein, as essential for seed germination to escape ABA-induced dormancy in *Arabidopsis* (**Figures 1** and **2**, Supplementary Figure S1). Interestingly, *ars1* mutants displayed higher ROS accumulation either in the absence or presence of ABA, indicating that ARS1 may act to repress ROS production (**Figures 3** and **4**). Furthermore, this finding also suggests that ARS1 may regulate the “oxidative window of germination” to prevent excess ROS accumulation during the period of breaking seed dormancy. Using *in silico* analysis, we found 211 C2H2-type zinc finger proteins that constitute the most abundant family of putative transcriptional regulators in *Arabidopsis*^3^. Several of these proteins act in abiotic stress responses, in particular ABA or oxidative stress signaling. SAZ (SA- AND ABA-DOWNREGULATED ZINC FINGER GENE), ZFP3 (ZINC FINGER PROTEIN 3), AZF1 (ARABIDOPSIS C2H2 ZINC FINGER PROTEIN 1), and AZF2 act as negative regulators in ABA signaling during seed germination and early seedling development (Jiang et al., 2008; Kodaira et al., 2011; Joseph et al., 2014). SAP12 (STRESS-ASSOCIATED PROTEIN 12) contains two AN1 zinc fingers and conformational changes due to redox states of cysteine residues located between the zinc finger structures modulate its activity (Ströher et al., 2009). ZAT10 (ZINC-FINGER OF ARABIDOPSIS 10) is phosphorylated by MPK3 and MPK6 and is involved in ROS-dependent ABA signaling (Mittler et al., 2006). The high conservation of functional motifs among the C2H2 zinc finger proteins suggests that these proteins may have similar molecular functions with respect to transcriptional regulation in diverse biological processes.

Reactive oxygen species act as important signaling molecules and control various biological processes including germination, growth, development, and abiotic stress responses. Diverse abiotic stresses lead to production of toxic levels of ROS, which causes oxidative damage to organelles such as chloroplasts and mitochondria in plant cells (Bailey-Serres and Mittler, 2006; Foyer and Noctor, 2009). To balance the accumulation of toxic ROS, plants have efficient, well-conserved mechanisms for the removal of ROS from cells, including both enzymatic and non-enzymatic ROS scavenging antioxidant systems (Mittler et al., 2004; Bailey-Serres and Mittler, 2006; Foyer and Noctor, 2009). For instance, SOD, CAT, and APX serve as ROS-scavenging enzymes. Abiotic stresses as well as ABA induce production of ROS such as oxygen radicals and hydrogen peroxide via plasma membrane-localized NADPH oxidases (Kwak et al., 2003). ROS accumulation in the *ars1* mutant plants may be caused by repressed expression of the SOD gene *CDS3* (**Figure 5**). Our results indicate that the function of ARS1 in stress tolerance may be associated with the regulation of antioxidant ability. However, the direct mechanisms by which ARS1 regulates *CDS3* expression are still elusive. To better understand the mechanisms, future research should examine the interaction between ARS1, ROS scavenging systems, and antioxidant enzyme activity.

Reactive oxygen species are generated in various subcellular organelles including chloroplasts, mitochondria, and peroxisomes, and they trigger changes in the nuclear transcriptome during stress (Apel and Hirt, 2004; Suzuki et al., 2012). ROS signaling occurs via interlinked exchanges between two distinct pathways: retrograde (organelle-to-nucleus) and anterograde (nucleus-to-organelle) signaling, which might be involved in acclimation, adaptation, or resistance against stresses (Suzuki et al., 2012). Disruption of ROS homeostasis can occur in chloroplasts or mitochondria, from which signals are transmitted to the nucleus via retrograde signaling cascades. We found that ARS1 in the nucleus was translocated to the cytoplasm upon exposure to ABA or oxidative stress, presumably in response to ROS signals (**Figure 6**). We cannot conclude that a protein is involved in retrograde regulation just because it exists in two different locations or relocalizes to another subcellular compartment depending on conditions. However, ARS1 translocates from the nucleus to cytoplasm in an ROS-dependent manner (**Figure 6**). This suggests that ARS1 may affect ROS-dependent anterograde signaling between the nucleus and cytoplasm under stress conditions or in response to ABA.

## Conclusion

The phytohormone ABA regulates important physiological processes, and is closely related with the accumulation of intracellular ROS to transfer signals triggered in diverse physiological and environmental cues. In this study, we isolated and designated an *ars1* mutant from large populations of *Arabidopsis* with T-DNA insertions as an ABA hypersensitive mutant. We identified that *ARS1* encodes a C2H2 type zinc finger domain protein and may play as a positive regulator for seed germination and maintenance of ROS homeostasis in response to ABA and oxidative stress, which trigger the induction of toxic ROS in cells, via the regulation of a gene for superoxide dismutase (*CSD3*). Interestingly, we also demonstrated that nuclear-localized ARS1 is translocated to the cytoplasm in response to ABA or oxidative stress. Translocation of ARS1 induced by ROS signals may help clarify the role of ROS-dependent anterograde signaling pathways that underlie plant stress responses. Taken together, our results suggest that ARS1 is essential to modulate seed germination and ROS homeostasis in response to ABA and oxidative stress in *Arabidopsis*.

## Author Contributions

DB, J-YC, and D-JY designed the experiments. DB performed most of the experiments, and J-YC and D-JY wrote the manuscript. DK, SL, and MK discussed and commented on the results and manuscripts. SK, BP, H-JL, HH, and HC performed some of the experiments. D-JY provided funding for research work as corresponding author.

## Conflict of Interest Statement

The authors declare that the research was conducted in the absence of any commercial or financial relationships that could be construed as a potential conflict of interest.

## Footnotes

1 http://bar.utoronto.ca/expressolog_treeviewer/

2 https://www.arabidopsis.org/

3 https://www.arabidopsis.org/
