# Supplementary material for: The Arabidopsis a zinc finger domain protein ARS1 is essential for seed germination and ROS homeostasis in response to ABA and oxidative stress
Source: Front Plant Sci. 2015 Nov 4;6:963. doi: 10.3389/fpls.2015.00963 (PMC4631831; doi:10.3389/fpls.2015.00963)
Supplement: Supplementary file 1 [file Data_Sheet_1.DOCX]

***Supplementary Material***

**Title: The *Arabidopsis* a zinc finger domain protein ARS1 is essential for seed germination and ROS homeostasis in response to ABA and oxidative stress**

Dongwon Baek^a,†^, Joon-Yung Cha^a,†^, Songhwa Kang^a^, Bokyung Park^a^, Hyo-Jung Lee^a^, Hyewon Hong^a^, Hyun Jin Chun^a^, Doh Hoon Kim^b^, Sang Yeol Lee^a^, Min Chul Kim^a^, Dae-Jin Yun^a,^*

* **Correspondence:** Corresponding Author:

E-Mail: [djyun@gnu.ac.kr](mailto:djyun@gnu.ac.kr)

^†^ These authors contributed equally to this work.

**1. Supplementary Figures and Tables**

**1. 1. Supplementary Figures**


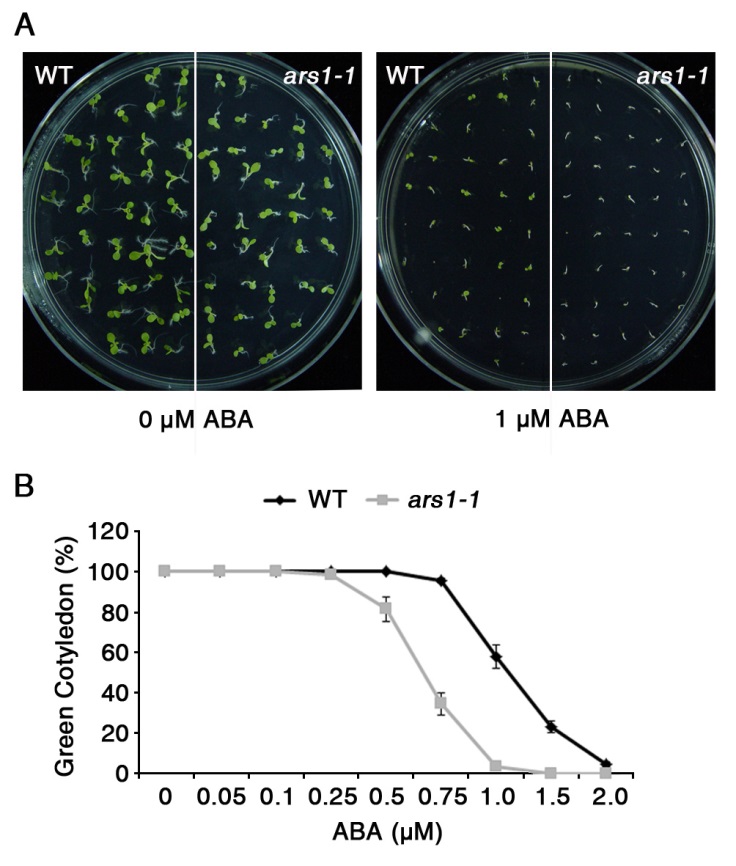


**Supplementary Figure S1.** The absence of ARS1 leads to ABA-hypersensitive seed germination. (**A**) Comparison of seed germination between the WT and *ars1-1* mutants exposed to 0 or 1 μM ABA. The photograph shows *Arabidopsis* seedlings after 5 d of ABA treatment. (**B**) Quantification of green cotyledons between WT and *ars1-1* mutants grown on various concentrations of ABA for 5 d. The data represent the means ± SE of three independent experiments, with 50 seeds per experiment.


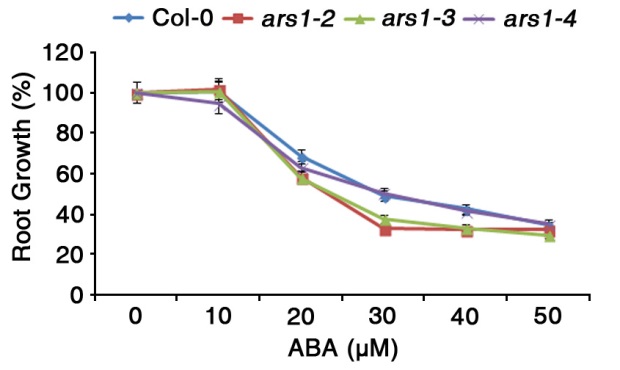


**Supplementary Figure S2**. Root elongation in *ars1* mutants exposed to ABA stress. Four-day-old WT Col-0 and *ars1* mutants on MS medium were transferred onto MS medium supplemented with various concentrations of ABA as indicated. The plates were observed 11 d after the transfer. The data represent the means ± SE of three independent experiments, with 10 seedlings per experiment.


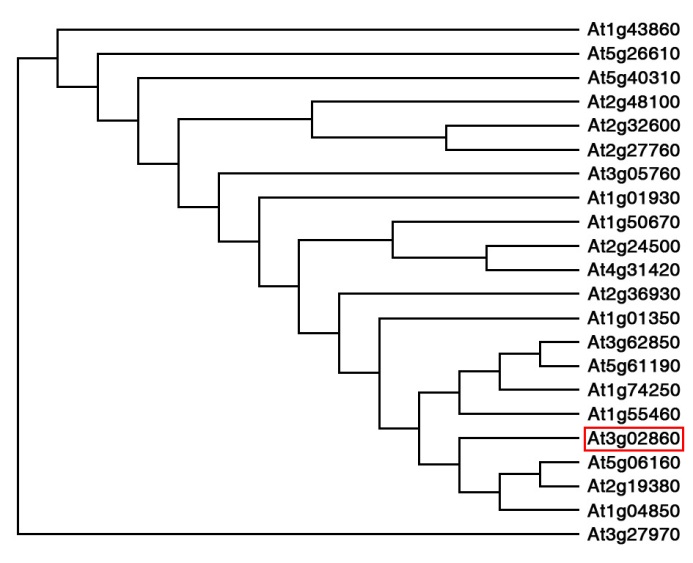


**Supplementary Figure S3**. Phylogenetic tree of ARS1 with evolutionary conserved subfamily of C3 subset, generated using the neighbor phylogenetic tree in BioEdit program. ARS1 (At3g02860) is marked as a *red box*.


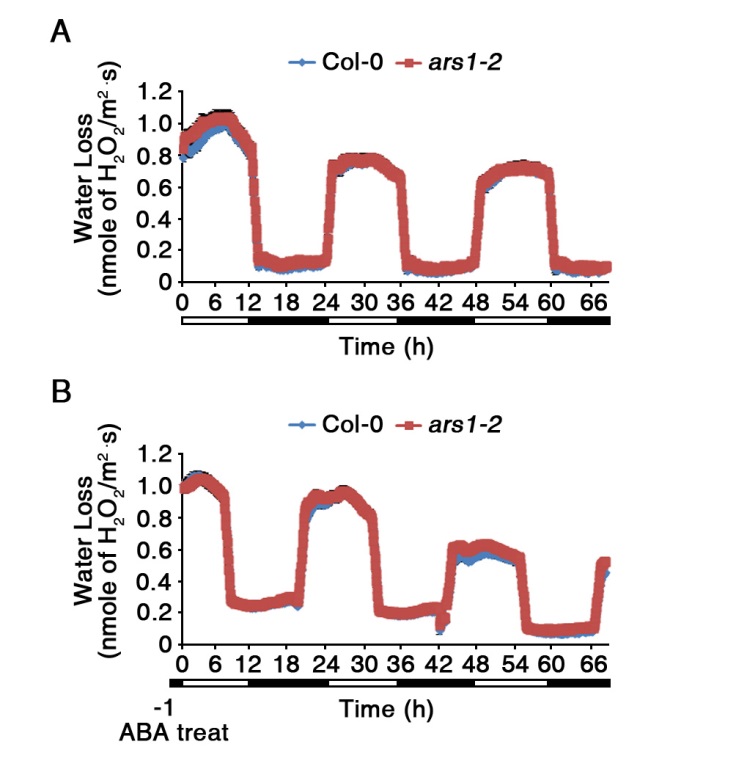


**Supplementary Figure S4**. Water loss assay. Leaves of 7-d-old WT Col-0 and *ars1-2* mutants grown on MS medium without (**A**) and with ABA (100 μM) for 1 h in dark (**B**) were excised and used for water loss assays. Data represent means ± SD of three independent experiments (10 to 12 measurements per point).


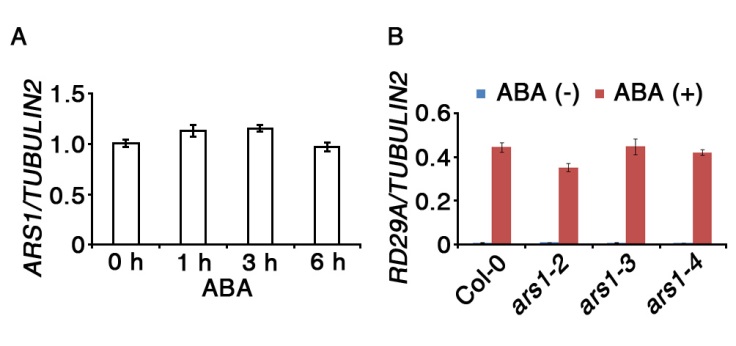


**Supplementary Figure S5**. Analysis of gene expression in response to ABA treatment. (**A**) Expression level of *ARS1* in response to ABA. Total RNA from 10-d-old seedlings of WT Col-0 treated with 100 μM ABA at the indicated time points were used for qRT-PCR analysis. (**B**) Expression levels of *RD29A* in *ars1* mutants after ABA treatment. Total RNA was isolated from 10-d-old seedlings of WT Col-0 and *ars1* mutants with or without ABA (100 μM) treatment for 3 h. Transcript levels were normalized to those of *TUBULIN2*. Bars represent mean ± SD of three biological replicates with three technical replicates each.


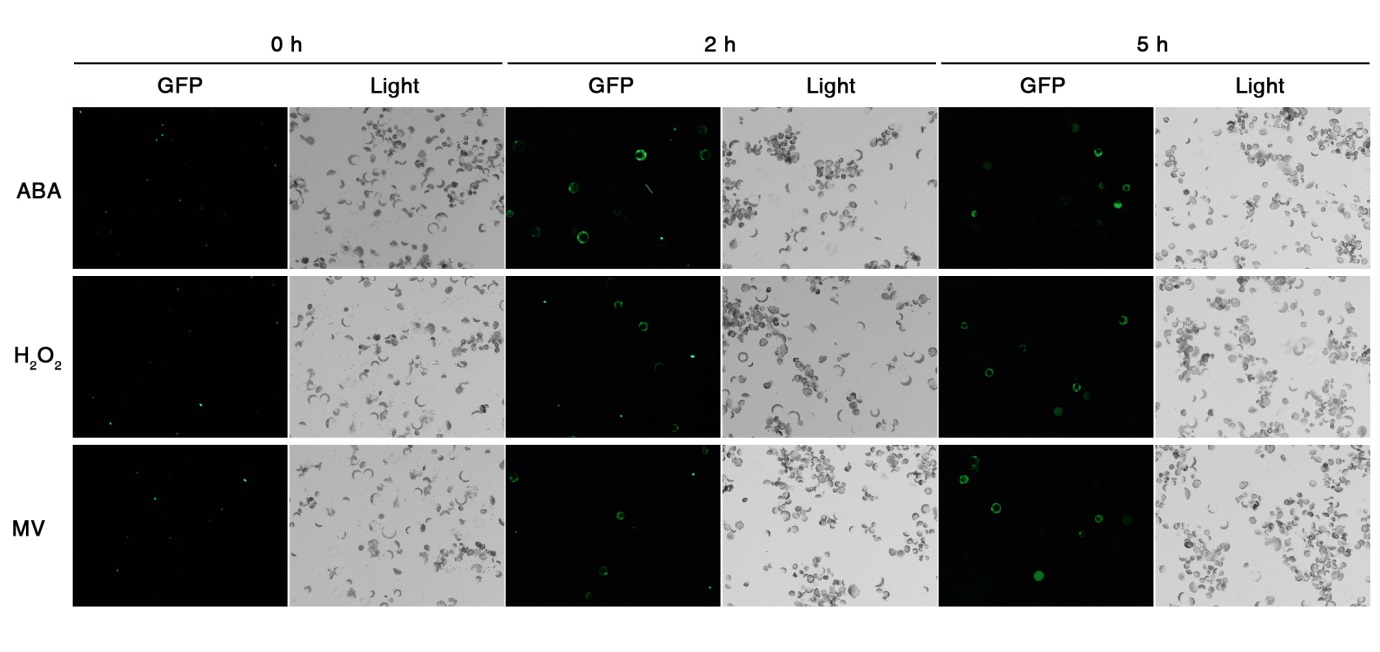


**Supplementary Figure S6**. ARS1 is exported from the nucleus to the cytoplasm in response to ABA and oxidative stress. Protoplasts were isolated from leaves of 3-week-old WT Col-0 plants transformed with *ARS1::sGFP*. Twelve hours after transformation, protoplasts were treated with ABA (100 μM), H_2_O_2_ (1.5 mM), or MV (3 μM) for the indicated times. Bars indicate 100 μm.

**1. 2. Supplementary Table**

**Supplementary Table S1.** List of primers used in this study.

| Primer Name | Sequence (5’-3’) | Purpose |
| --- | --- | --- |
| LB1 | ATACGACGGATCGTAATTTGT | TAIL-PCR |
| LB2 | TAATAACGCTGCGGACATCTAC | TAIL-PCR |
| LB3 | TTGACCATCATACTCAT TGCTG | TAIL-PCR |
| DP1 | NTCGASTWTSGWGTT | TAIL-PCR |
| DP2 | NGTCGASWGANAWGAA | TAIL-PCR |
| DP3 | WGTGNAGWANCANAGA | TAIL-PCR |
| ARS1-RT-F | ATGGATGCTCAAGCGAAG | RT-PCR |
| ARS1-RT-R | AAGATGTTGAGCTCT | RT-PCR |
| TUBULIN2-RT-F | CGTGGATCACAGCAATACAGAGCC | RT-PCR |
| TUBULIN2-RT-R | CCTCCTGCACTTCCACTTCGTCTTC | RT-PCR |
| ARS1-F-XbaI | GCTCTAGAATGGATGCTCAAGCGAAG | Plasmid construction |
| ARS1-R-BamHI | GCGGATCCAAGATGTTGAGCTCT | Plasmid construction |
| TUBULIN2-qRT-F | TGGCATCAACTTTCATTGGA | qRT-PCR |
| TUBULIN2-qRT-R | ATGTTGCTCTCCGCTTCTGT | qRT-PCR |
| ARS1-qRT-F | CCTGAAGTGATCGATGCACCAG | qRT-PCR |
| ARS1-qRT-R | TGGTGTAATCGGAAGACACGAC | qRT-PCR |
| RD29A-qRT-F | CCTGAAGTGATCGATGCACCAG | qRT-PCR |
| RD29A -qRT-R | TGGTGTAATCGGAAGACACGAC | qRT-PCR |
| CCS-qRT-F | CCTCTACCAACCCCAAATCTC | qRT-PCR |
| CCS -qRT-R | GATCAGAAGTGAGAGCAGTCG | qRT-PCR |
| CDS3-qRT-F | TCCGAGGCTGTCTTCAATTC | qRT-PCR |
| CDS3 -qRT-R | GAAGTGAGGTCCAGTAGAGATG | qRT-PCR |
| APX1-qRT-F | TGACATTCCTTTCCACCCTG | qRT-PCR |
| APX1 -qRT-R | CTTGGTAGCATCAGGAAGACG | qRT-PCR |
| APX2-qRT-F | TTGCTGTTGAGATCACTGGAG | qRT-PCR |
| APX2 -qRT-R | TCCGACCAAACACATCTCTTAG | qRT-PCR |
